# Proteogenomic Analysis Identifies a Novel Human SHANK3 Isoform

**DOI:** 10.3390/ijms160511522

**Published:** 2015-05-19

**Authors:** Fahad Benthani, Phuong N. Tran, Nicola Currey, Irvin Ng, Marc Giry-Laterriere, Louise Carey, Maija R. J. Kohonen-Corish, Laurent Pangon

**Affiliations:** 1 The Kinghorn Cancer Centre, Garvan Institute of Medical Research, Sydney, NSW 2010, Australia; E-Mails: f.benthani@garvan.org.au (F.B.); p.tran@garvan.org.au (P.N.T.); n.currey@garvan.org.au (N.C.); i.ng@garvan.org.au (I.N.); m.giry-laterriere@garvan.org.au (M.G.-L.); 2 St Vincent’s Clinical School, UNSW Medicine, UNSW Australia, Sydney, NSW 2052, Australia; 3 Sydney Genome Diagnostics, the Children’s Hospital at Westmead, Sydney, NSW 2145, Australia; E-Mail: louise.carey@health.nsw.gov.au

**Keywords:** proteogenomic, SHANK3, MCC

## Abstract

Mutations of the *SHANK3* gene have been associated with autism spectrum disorder. Individuals harboring different *SHANK3* mutations display considerable heterogeneity in their cognitive impairment, likely due to the high *SHANK3* transcriptional diversity. In this study, we report a novel interaction between the Mutated in colorectal cancer (MCC) protein and a newly identified SHANK3 protein isoform in human colon cancer cells and mouse brain tissue. Hence, our proteogenomic analysis identifies a new human long isoform of the key synaptic protein SHANK3 that was not predicted by the human reference genome. Taken together, our findings describe a potential new role for MCC in neurons, a new human SHANK3 long isoform and, importantly, highlight the use of proteomic data towards the re-annotation of GC-rich genomic regions.

## 1. Introduction

*SHANK3* has been described as one of the most promising candidate susceptibility genes for autism and autism spectrum disorders [1,2,3]. Heterozygous deletion or mutation of *SHANK3* is thought to be the cause of core neurodevelopmental and neurobehavioral deficits in the 22q13 deletion syndrome, Phelan-McDermid syndrome, an autism spectrum disorder with developmental delay, absent or delayed speech and mild facial dysmorphism [4]. Importantly, a recent study also shows that *Shank3* mutant mice display autistic-like behaviors and striatal dysfunction [5].

SHANK3 is a large protein with a well defined PDZ-domain that binds several effector proteins such as βPIX, receptor tyrosine kinase RET9, latrophilin, l-type Ca^2+^ channels CaV1.3a C, mGluR and GKAP [6,7,8,9,10,11,12,13,14].

*Mutated in colorectal cancer* (*MCC*) is a tumour suppressor gene in colon and liver tissues [15,16,17]. MCC is a 829 amino acids protein with a well defined Class-I PDZ binding motif (ETSL) that binds to the PDZ-domain containing protein SCRIB [18,19]. Several functions have been attributed to MCC in epithelial cells including DNA damage response [20], transcriptional regulation [21,22,23], lamellipodia formation and cell migration [19]. Despite its high expression level in the brain [24] no functional study in the neural tissue has been performed. However, a recent study identified *MCC* as a potential candidate gene for autism spectrum disorders [25].

In this study, we used a proteogenomic approach to identify a novel SHANK3 isoform that binds to MCC.

## 2. Results

### MCC Interacts with a Newly Characterized Human SHANK3 Isoform

We recently reported that MCC is enriched at the migratory edge of colon epithelial cells and binds to the PDZ-domain containing protein SCRIB [19]. In order to identify novel MCC interacting partners we undertook a series of co-immunoprecipitation assays using endogenous MCC as bait, followed by mass spectrometric analyses. Endogenous MCC was immunoprecipitated from sub-confluent human colon cancer cell line SW480. We narrowed down our search on proteins containing PDZ domains.

**Figure 1:**
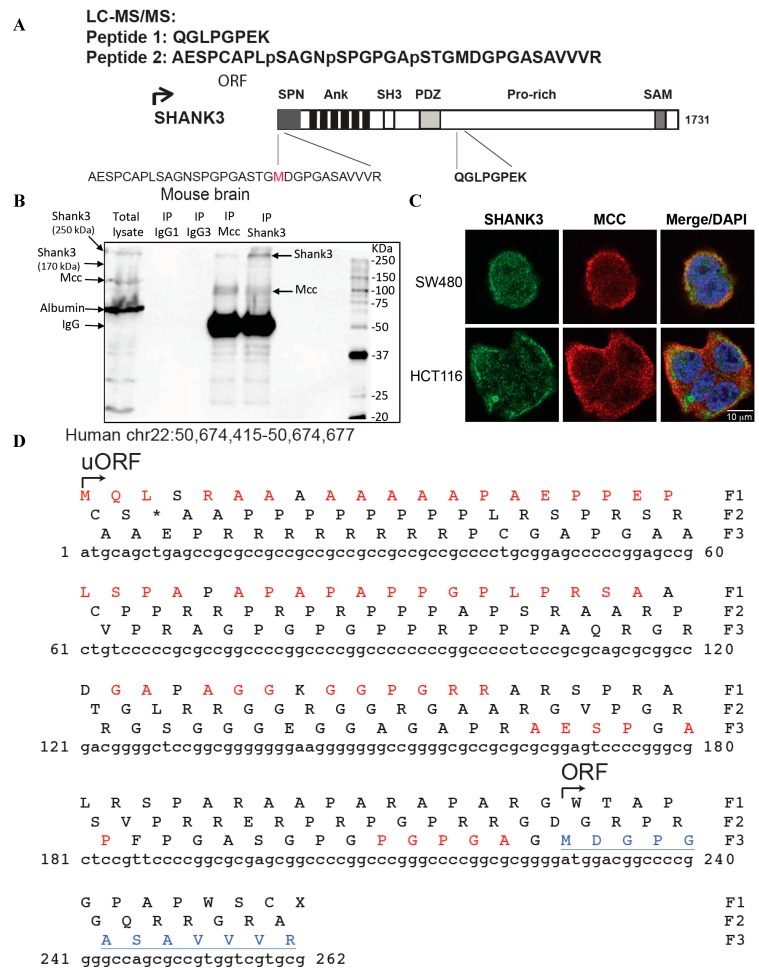

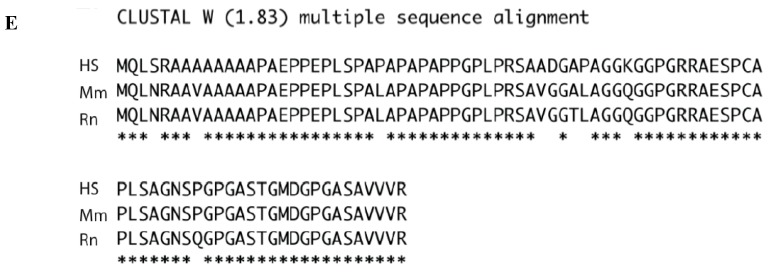
Identification of a new human SHANK3 isoform. (**A**) LC-MS/MS identifies a SHANK3 tryptic peptide (Peptide 2) corresponding to a novel human SHANK3 isoform previously only identified in mouse and rat. Endogenous MCC was immunoprecipitated from SW480 human colon cancer cells. The immunoprecipitate was processed for LC-MS/MS and the data searched using Mascot. Among the tryptic peptides identified, two peptides corresponded to the SHANK3 protein; (**B**) Endogenous Mcc interacts with endogenous Shank3 in mouse brain tissue. Endogenous Shank3 and Mcc were immunoprecipitated (IP) from mouse brain tissue. Lysates and immunoprecipitates were run and blotted for Shank3 and Mcc; (**C**) Confocal microscopy for paraformaldehyde-fixed SW480 and HCT116 cells probed with anti-SHANK3 (green) and anti-MCC antibodies (red) show partial cell membrane colocalisation; (**D**) Shown is the region of the human genome sequence encompassing chr22:50,674,415 to chr22:50,674,677 that was extracted from UCSC Dec 2013. GRCh38/hg38. ORF: Open Reading Frame; uORF: upstream Open Reading Frame. Translation of chr22:50,674,415 to chr22:50,674,677 using three open reading frames. Amino acids in red are found in the mouse or rat Shank3 protein. The predicted new *N*-terminal amino acids sequence is derived from the first 54aa of Frame 1 (F1) followed by the second tryptic peptide. The beginning of the known translated region is underlined in blue; (**E**) Multiple alignment of mouse Shank3, rat Shank3 and predicted human SHANK3 was done using the software ClustalW2.1. Asterisks indicate conservation between mouse, rat and the predicted human sequence.

Two tryptic peptides matching the PDZ domain containing protein SHANK3 were identified (Figure 1A). Remarkably, peptide-2, a 33-amino acid tryptic peptide, indicated that translation of this SHANK3 isoform started upstream of the known Open Reading Frame (ORF) located at chr22:50,674,641 (UCSC Dec 2013. GRCh38/hg38) (http://genome.ucsc.edu/) [26]. Hence, despite this particular isoform being previously identified in mouse (XP_006521283.1) and rat (XP_006242301.1), it had not been identified or predicted in humans. Consistent with reported Mcc expression in the brain [24], we confirmed endogenous Mcc binding with endogenous Shank3 protein in mouse brain tissue with co-immunoprecipitation (Figure 1B). Confocal microscopy also showed that endogenous MCC partially co-localizes with endogenous SHANK3 at the cell membrane of SW480 and HCT116 colon cancer cells (Figure 1C).

Analysis of the human reference genome (RefSeq, UCSC) revealed a potential upstream Open Reading Frame (uORF) located in SHANK3 5' UTR transcript (Ensembl: ENST00000414786) and an extremely high GC-rich region upstream of the known ORF (ORF) (Figure 1D) [27]. GC rich regions are known to be prone to sequencing errors. Translation of the predicted uORF transcript, GRCH38/hg38 chr22:50674415-50674677, is shown in Figure 1D. Remarkably, 46 out of 54 amino acids from the first open reading frame (shown in red) are identical to the mouse and rat Shank3 proteins (XP_006521283.1 and XP_006242301.1 respectively). Furthermore, translation product from the third open reading frame (Frame-3) (residues 55 to 75) is partially consistent with the SHANK3 tryptic peptide-2 (shown in red) and in frame with the known human SHANK3 ORF (residues from the known ORF are underlined).

Multiple alignments between the mouse protein (GRCm38/mm10; chr15:89499632-89499892; XP_006521283.1), the rat protein (RGSC 5.0/rn5; chr7:130159261-130159521; XP_006242301.1) and translation product from the newly predicted uORF human isoform, show a high degree of identity (91% and 88% identity between human/mouse and human/rat sequences respectively) (Figure 1E). Despite several attempts, we were unable to PCR amplify or sequence this region likely due to the high GC-rich sequence.

Taken together, based on the mouse sequence, rat sequence, and our proteogenomic data we propose the existence of a new human SHANK3 isoform (UCSC genome browser GRCh38/hg38 chr22:50,674,415–50,674,641, MQLSRAAAAAAAAPAEPPEPLSPAPAPAPAPPGPLPRSAADGAP AGGKGGPGRRAESPCAPLSAGNSPGPGASTG) that adds 75 amino acids to the *N*-terminal part of the protein.

## 3. Discussion

Our study unveils a novel human *SHANK3* transcript that adds to the already complex *SHANK3* transcript diversity. Hence, several splice variants of *SHANK3* with alternative translational start and stop codons have been reported, suggesting that the SHANK3 protein interactions are regulated by alternative splicing and alternative promoter usage [28,29,30]. The origin of these SHANK3 isoforms remains unclear and could arise from alternative transcription start sites or alternative translation start sites as previously reported for SHANK1 [31].

Importantly, a recent study comprehensively characterized a number of *Shank3* transcripts in mice and highlighted the relation between the large number of *Shank3* transcripts and the phenotypic heterogeneity caused by the different *SHANK3* mutations in humans [32,33]. Here we show that in the mouse brain, Mcc binds to this longer Shank3 isoform (approximately 250 kDa). We also noticed that the shorter Shank3 isoform (170 KDa) is less abundant than the long isoform and only detectable following Shank3 immunoprecipitation. The functional role of this 75aa *N*-terminal domain is yet unknown but previous work has shown that different isoforms of Shank3 are temporally and spatially specific and are differently regulated by neural activity [33].

In view of the role of SHANK3 in neurodevelopmental, neurobehavioral and autism spectrum disorders, future work to decipher the functional role of this 75aa *N*-terminal domain and its interaction with MCC is now warranted. Remarkably, another recent study identified MCC as a potential candidate gene for autism spectrum disorders [25]. Importantly, this work also reinforces the utility of proteogenomics to help identify new transcripts and inaccuracies in genomic reference sequences.

## 4. Experimental Section

### 4.1. Co-Immunoprecipitation, Mass-Spectrometry

Endogenous MCC was immunoprecipitated using mouse monoclonal antibody (#10740, BD Biosciences, Franklin Lakes, NJ, USA) from SW480 colon cancer cell line and processed for mass-spectrometry. MCC or control immunoprecipitates were separated using 10% SDS-PAGE and the SYPRO-stained band was excised and destained in 1 mL of 50% acetonitrile and 200 mM ammonium bicarbonate at room temperature for 45 min with shaking. All solutions were carefully removed prior to the addition of modified trypsin (12.5 ng/μL) in 100 mM NH4HCO3 and incubation overnight at 37 °C. Peptides were extracted by the addition of 0.1 mL of 5% formic acid and incubation at 37 °C for 1 h. Peptides were further extracted by the addition of 0.1 mL of 100% acetonitrile and incubation at 37 °C for 1 h. The entire supernatant was then vacuum-dried. The peptides were redissolved in 20 μL of 5% formic acid for LC-MS/MS analysis as previously described [20]. Data were analyzed using Mascot server version 2.2 against the entire International Protein Index (IPI) database. The settings used for the Mascot search were as follows: two missed cleavages were allowed; enzyme was trypsin cleaving after arginine and lysine; variable modifications were methionine oxidation, propionamide cysteine, and phosphorylation of serine, threonine, or tyrosine; no fixed modifications were used; a mass tolerance of 6 ppm was used for precursor ions; and a MS/MS tolerance of 0.5 Da was used for fragment ions. False recovery rate was less than 1% and localization score cut-off was greater than 75%.

### 4.2. Co-Immunoprecipitation and Western Blotting

Shank3 and Mcc were immunoprecipitated from mouse brain tissue. Immunoprecipitates were run on a 10% SDS-PAGE gel before being blotted with the appropriate antibody as labeled. Antibodies used were mouse anti-Shank3 (75–109, UC Davis/NIH NeuroMab Facility, Davis, CA, USA), and mouse anti-MCC (#610740, BD Biosciences). This was repeated on two more harvested mouse brain tissues for confirmation.

### 4.3. Immunofluorescence Microscopy

Epithelial colon cancer cell lines SW480 and HCT116 were cultured in RPMI media supplemented with 10% FBS on glass coverslips. Cells were then fixed with fresh 2% *v*/*v* paraformaldehyde in phosphate buffered saline (PBS) for 20 min at room temperature (RT) and washed with PBS, before being permeabilized in 0.5% *v*/*v* Triton X-100 (Sigma, St. Louis, MO, USA) in PBS for 10 min. The cells were then blocked with 3% BSA. Labeling was performed in PBS containing 3% BSA and diluted primary antibodies for 2 h at RT. Primary antibodies used were: rabbit anti-Shank3 (1:500, Santa Cruz Biotec, Dallas, TX, USA) and mouse anti-MCC (1:500, BD Biosciences). The cells were then incubated with secondary antibodies conjugated to either Alexa Fluor 488 (green) or Cy3 (red) dyes (Life Technologies, Grand Island, NY, USA), for at least 1 h. DNA was stained with 0.1 μg/mL 4',6-diamidino-2-phenylindole (DAPI). Cells were mounted on slides using Dako anti-fading mounting media. Optical sections were analyzed by confocal microscopy (DMI 6000 SP8, Leica, Wetzlar, Germany) using a 63x/1.4 NA objective lens. Color, brightness and contrast were adjusted with ImageJ 1.48v software (NIH, Bethesda, MD, USA) for clarity.
